# Chitin-deacetylase activity induces appressorium differentiation in the rice blast fungus *Magnaporthe oryzae*

**DOI:** 10.1038/s41598-017-10322-0

**Published:** 2017-08-29

**Authors:** Misa Kuroki, Kana Okauchi, Sho Yoshida, Yuko Ohno, Sayaka Murata, Yuichi Nakajima, Akihito Nozaka, Nobukiyo Tanaka, Masahiro Nakajima, Hayao Taguchi, Ken-ichiro Saitoh, Tohru Teraoka, Megumi Narukawa, Takashi Kamakura

**Affiliations:** 1 Tokyo University of Science, Department of Applied Biological Science, Faculty of Science and Technology, 2641, Yamazaki, Noda, Chiba 278-8510 Japan; 2 Nagoya University, Graduate School of Bioagricultural Sciences, School of Agricultural Sciences, Furo-cho, Chikusa, Nagoya, Aichi 464-8601 Japan; 3 Tokyo University of Agriculture and Technology, University Research Administration Center, 2-24-16, Naka-cho, Koganei, Tokyo 184-8588 Japan; 4 Tokyo University of Agriculture and Technology, Institute of Symbiotic Science and Technology, 3-5-8, Saiwai-cho, Fuchu, Tokyo 183-8509 Japan

## Abstract

The rice blast fungus *Magnaporthe oryzae* differentiates a specialized infection structure called an appressorium to invade rice cells. In this report, we show that *CBP1*, which encodes a chitin-deacetylase, is involved in the induction phase of appressorium differentiation. We demonstrate that the enzymatic activity of Cbp1 is critical for appressorium formation. *M. oryzae* has six CDA homologues in addition to Cbp1, but none of these are indispensable for appressorium formation. We observed chitosan localization at the fungal cell wall using OGA^488^. This observation suggests that Cbp1-catalysed conversion of chitin into chitosan occurs at the cell wall of germ tubes during appressorium differentiation by *M. oryzae*. Taken together, our results provide evidence that the chitin deacetylase activity of Cbp1 is necessary for appressorium formation.

## Introduction

The worldwide rice harvest is approximately 500 million tonnes per year^1^, but 10–15% of this total yield is lost due to disease^2^. One of the major causes of disease losses in rice is the fungus *Magnaporthe oryzae*, which causes rice blast. Rice leaves infected by *M. oryzae* display necrotic lesions and heavy infections can kill rice seedlings. The infection process of *M. oryzae* is initiated by the attachment of a conidium to the rice surface through the release of spore tip mucilage^3^. After germination in a water droplet on the rice leaf, the conidium elaborates a germ tube^4^, which begins to form a dome-shaped appressorium at its tip^5–7^. The fungus then generates enormous turgor pressure in the appressorium and produces a penetration peg that pierces the plant cell wall to enter an epidermal cell^8, 9^. The appressorium is a specialized infection structure that is crucial for host plant penetration^10^. Therefore, knowledge of the mechanism of appressorium formation may contribute to disease control.

Appressorium development can be induced by several physical and chemical factors; surface hydrophobicity^11, 12^, the hardness of the contact surface^13, 14^ and cutin monomers released from the plant surface^15^. Signal transduction pathways such as cAMP signalling and mitogen-activated protein kinase cascades are required for appressorium morphogenesis^6, 10^.

Kamakura *et al*.^16^ previously reported that the gene *CBP1*, which encodes a Chitin-Binding Protein (MGG_12939.7), is involved in appressorium formation by *M. oryzae*. *CBP1* is specifically expressed in germlings and the null mutant of *CBP1* shows delayed appressorium differentiation on hydrophobic surfaces. Eight hours post inoculation (hpi), the frequency of appressorium formation in the Δ*cbp1* mutant was very low, but increased to the same level as in an isogenic wild type strain at 24 hpi on hydrophobic artificial substrates. The Δ*cbp1* mutant also retains the ability to cause disease symptoms on leaves of susceptible rice cultivars^16^. When conidia of Δ*cbp1* were treated with 3-isobutyl-1-methylxanthine, which induces intracellular accumulation of cAMP^17^, or 1,16-hexadecanediol (HDD), a minor component of cutin^15^, they were able to form appressoria on artificial hydrophobic surfaces at 8 hpi, as similarly to the wild type strain^16^. These results suggest that Cbp1 is not essential for appressorium formation and primary infection of rice leaves, but clearly indicate that Cbp1 facilitates appressorium differentiation on artificial substrates. It was predicted that Cbp1 has a signal peptide sequence and Ser/Thr cluster and that the protein localizes at the fungal cell surface^16^. Recently, Geoghegan and Gurr^18^ observed cell-surface localization of Cbp1 through expression of a Cbp1-mCherry fusion protein. Cbp1 localizes to the periplasm in a similar way to the sensor of hydrophobicity Mpg1^19^ and the MagB heterotrimeric G-protein catalytic subunit^15^. Furthermore, the amino acid sequence of Cbp1 suggests it is a glycosyl phosphatidyl inositol (GPI)-anchored protein. Some GPI-anchored proteins are involved in signalling pathways, such as Ecm33 in *Aspergillus fumigatus* ^20^, and Pga31 and Pga62 in *Candida albicans* ^21^. These characteristics of Cbp1 support a potential role for the protein as an upstream effector of a signal transduction pathway.

In the present work, we show that Cbp1 actually acts as a chitin-deacetylase (CDA) (consistent with the findings of^18^). CDA generally converts chitin into chitosan. Chitin is a linear β-(1,4)-linked insoluble homopolymer of the acetylated amino sugar *N*-acetylglucosamine (GlcNAc). Chitin is a major component of the cells of most filamentous fungi^22^ and contributes to the strength and integrity of the fungal cell wall and septum^23^. Chitosan, generated by deacetylation of chitin, is produced enzymatically by CDA, which hydrolyses the *N*-acetamido groups of GlcNAc residues in chitin^24^. CDA is not currently known to act as part of a signalling pathway in other species, so we investigated whether Cbp1 acts by a novel mechanism. Before now, CDA activity has only been observed indirectly and enzymatic CDA activity has not been observed in *M. oryzae*.

## Results

### CDA activity of Cbp1 is required for appressorium formation

**Figure 1:**
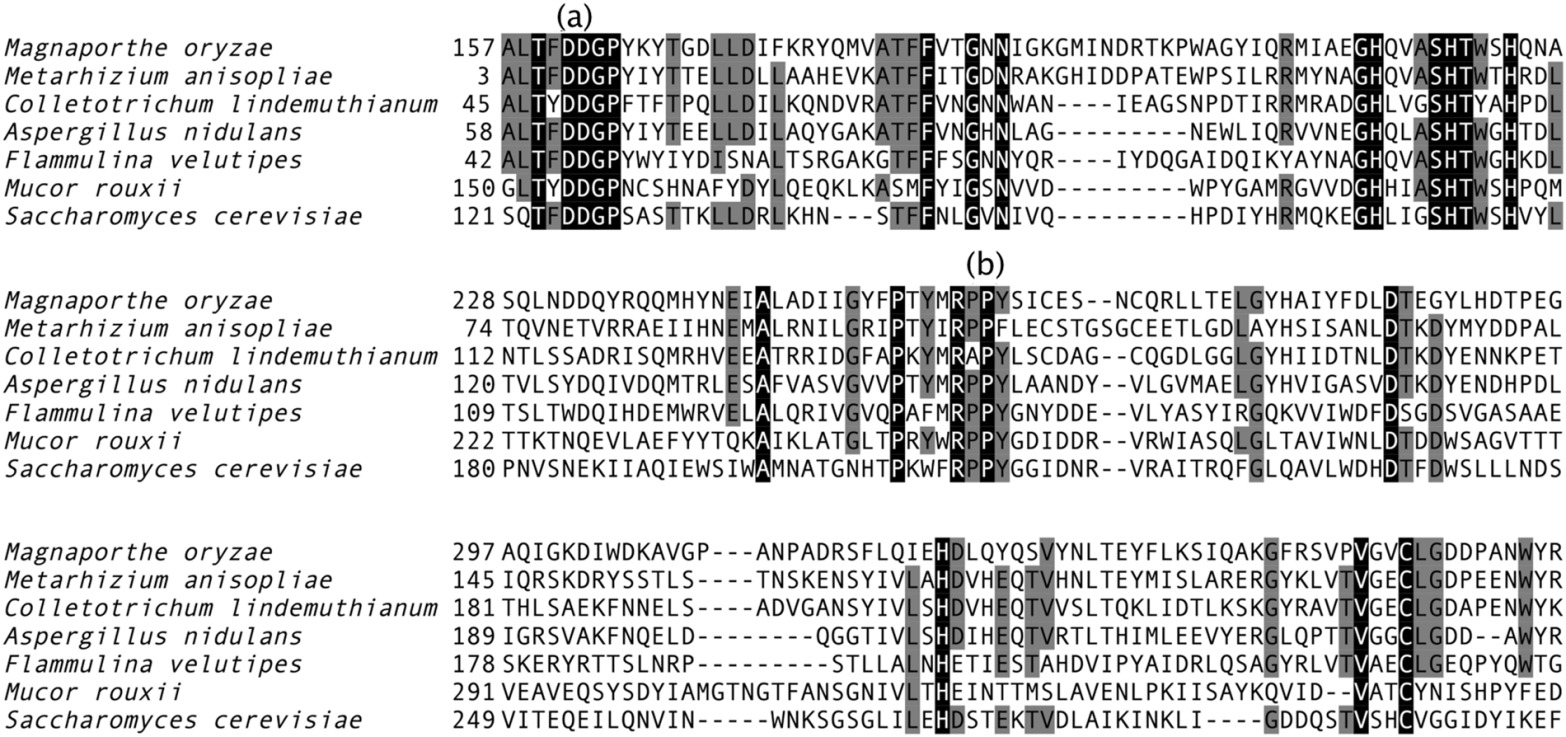
Multiple alignment of protein sequences. The amino acid sequence of the CDA-homologous domain in Cbp1 was aligned with CDAs in other species. The CDA sequences included (GenBank accession number) KFG84684.1 from *Metarhizium anisopliae*, AAT68493.1 from *Colletotrichum lindemuthianum*, ACF22100.1 from A*spergillus nidulans*, BAE92728.1 from *Flammulina velutipes*, CAA79525.1 from *Mucor rouxii*, and KZV09551.1 from *Saccharomyces cerevisiae*. White letters on a black background indicate residues perfectly conserved across all the sequences, black letters on a grey background indicate regions conserved in at least five species out of seven. (**a**) Is a group of amino acids (TFDD) that comprise the CDA active site, including two aspartate residues. (**b**) Is also part of the CDA active site; RPPY, including tyrosine.

CDA genes have previously been cloned and characterized from several fungi and insects. Multiple sequence alignments have demonstrated that they have five well-conserved catalytic motifs^25^. These motifs make up the CDA active site^26^ and are conserved in CDA genes such as those in *Metarhizium anisopliae* [KFG84684.1]^27^, *Colletotrichum lindemuthianum* [AAT68493.1]^28^, *Aspergillus nidulans* [ACF22100.1]^29^, *Flammulina velutipes* [BAE92728.1]^30^, *Mucor rouxii* [CAA79525.1]^24^ and *Saccharomyces cerevisiae* [KZV09551.1]^31^ (Figs 1 and S1). These proteins have been demonstrated to show CDA activity experimentally. We observed that Cbp1 possessed these motifs. To test whether Cbp1 had CDA activity, we generated CDA active site substitution mutants and observed their phenotypes. One of the conserved motifs (TFDD, Fig. 1(a)) includes two aspartic acid residues; one was reported to interact with zinc or cobalt and the second binds acetate released from the substrate^26^.

**Figure 2:**
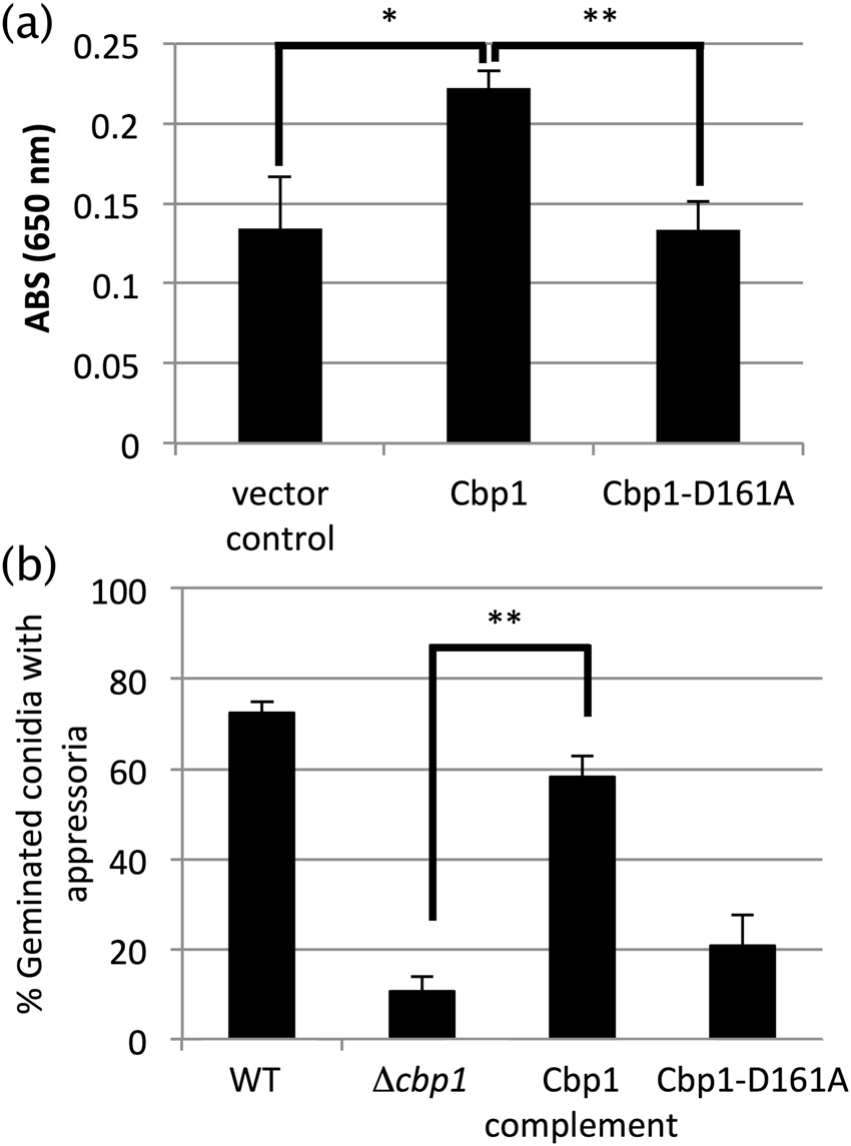
Effect of point mutation of Cbp1 on CDA activity and appressorium formation. Cbp1 and Cbp1-D161A were compared for CDA activity and appressorium formation. Cbp1-D161A has a point mutation changing the first aspartate residue in the (**a**) region in Fig. 1 to alanine. (**a**) The CDA activity of crude extract expressed in *E. coli* was observed by the MBTH method. The experiment was performed in triplicate for each sample and repeated four times. *p < 0.05, **p < 0.01 (Student’s *t*-test) compared with Cbp1. Error bars indicate standard error. (**b**) Appressorium formation rates were scored at 6 h post inoculation (hpi) on hydrophobic polyvinyl chloride (PHOB-PC). The experiment was performed in triplicate for each sample and repeated three times. Appressorium formation rates were calculated by dividing the number of conidia with appressoria by the number of germinated conidia. **p < 0.01 (Student’s *t*-test) compared with Δ*cbp1*. Error bars indicate standard error.

We chose the first aspartate residue in the (a) domain and replaced it with alanine. We used the pCold I vector and heterologously expressed Cbp1 and the Cbp1-D161A substitution mutant in *Escherichia coli*. The crude extract of the Cbp1-expression strain showed significantly higher CDA activity than those of the vector control and Cbp1-D161A-expression mutant (Fig. 2(a)). These data indicated that Cbp1 has activity as a CDA.

We then transformed Δ*cbp1 M. oryzae* using Cbp1-D161A and obtained a CDA-inactive Cbp1 mutant. We concurrently generated a wild type *CBP1*-complemented strain. The ability to elaborate appressoria was tested in these mutants and the wild type strain. In the *CBP1*-complemented strain, appressorium formation levels were significantly increased compared with the Δ*cbp1* mutant. By contrast, in the inactive mutant Cbp1-D161A, appressorium formation appeared the same as in the Δ*cbp1* mutant (Fig. 2(b)). We similarly generated substitution mutants of the second aspartate residue in the (a) domain and another motif (RPPY, Fig. 1(b)), and observed similar phenotypes (Fig. S2). These results suggested that the CDA activity of Cbp1 plays an important role in appressorium formation on a solid surface made of hydrophobic polyvinyl chloride (PHOB-PC).

### Other CDA genes could not fully complement the function of Cbp1

**Figure 3:**
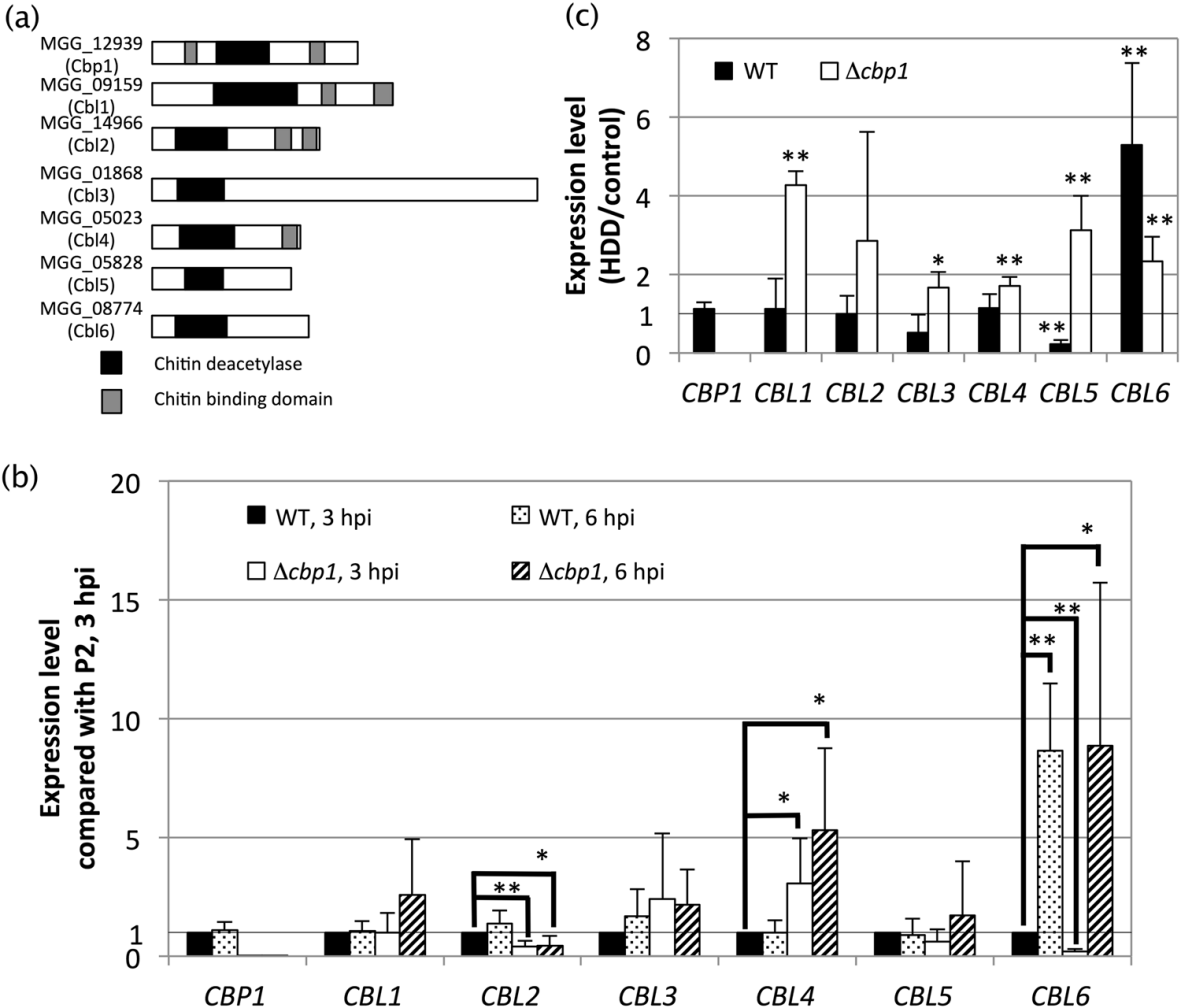
Analyses of CDA homologous proteins. (**a**) The domain features of seven CDA-homologous proteins. Black boxes indicate homology to CDA, dark grey boxes indicate chitin binding domains. (**b**) and (**c**) show transcriptomic analyses of the seven CDA-homologous genes. The expression levels of *CBP1* and the other CDA-homologous genes were detected by real-time PCR. Each cDNA was reverse transcribed from RNA extracted from germinated conidia (3 hpi) or appressoria (6 hpi). The expression levels were normalized by the expression level of *HPRT* in each strain. Real-time PCR experiments were repeated at least four times from each RNA preparation to confirm reproducibility. (**b**) Time-lapse analysis at 3 and 6 hpi. The expression values for each gene were normalized to the expression in the WT at 3 hpi. Expression levels <1 indicate lower expression than in the WT at 3 hpi and levels >1 indicate higher expression than in the WT at 3 hpi. *p < 0.05, **p < 0.01 (Student’s *t*-test) compared with the WT at 3 hpi. Error bars indicate standard deviation. (**c**) Effect of 1,16-hexadecanediol (HDD). Expression levels are shown as ratios compared with the expression level in each strain without HDD as a control. Numerical values <1 indicate downregulation and values >1 indicate upregulation by HDD addition. *p < 0.05, **p < 0.01 (Student’s *t*-test) compared with each control. Error bars indicate standard deviation.

*M. oryzae* has seven homologous CDA genes including *CBP1* ^32^. We reasoned that the other gene(s) might compensate for a loss of Cbp1 function. We therefore compared their sequences using CLUSTAL W, but they did not share strong homology with each other. We focused on two Cbp1 like proteins, MGG_09159 (chitin-deacetylase, which we named Cbp1 like protein 1, Cbl1) and MGG_14966 (a hypothetical protein, which we named Cbl2), which resembled Cbp1 at the domain level (Fig. 3(a)). Cbl1 and Cbl2 shared two chitin-binding domains similar to those of Cbp1, but their positions were different from those in Cbp1.

We compared the expression patterns of the seven CDA homologues between the wild type and Δ*cbp1* at 3 and 6 hpi (Fig. 3(b)). Differentiation of appressoria was initiated in the wild type at 3 hpi and in Δ*cbp1* at 6 hpi. In the wild type, *CBL6* was upregulated at 6 hpi compared with 3 hpi but the expression of the other genes did not change between 3 and 6 hpi. In Δ*cbp1*, *CBL2* was downregulated and *CBL4* was upregulated regardless of time point. Interestingly, in Δ*cbp1*, *CBL6* was downregulated at 3 hpi and upregulated at 6 hpi. These results indicated that *CBL6* might act in a later phase of appressorium differentiation and that *CBL4* was upregulated by *CBP1* deletion, but could not completely compensate for the loss of Cbp1 function.

At 6 hpi after adding HDD, appressorium formation in Δ*cbp1* occurred at the same rate as in the wild type. Thus, we compared the expression patterns of the seven CDA homologues at 3 hpi with or without HDD (Fig. 3(c)). In the wild type, *CBL5* was downregulated and *CBL6* was upregulated by adding HDD. In Δ*cbp1*, all genes except *CBL2* were upregulated by adding HDD, and *CBL2* also appeared to be upregulated although the change was not significant. These results suggested that the upregulation of all *CBL*s may compensate for the deletion of *CBP1*.

We next investigated the role of Cbl1 in appressorium development in more detail. The *cbl1* mutant strain did not show any visible defects in colony growth or morphology during appressorium differentiation. However, a Δ*cbp1*Δ*cbl1* double mutant strain showed a decrease in conidiation, conidial adhesion, and appressorium formation (Fig. S3). These results suggested that Cbl1 plays a minor role in appressorium formation at 6 hpi and acts synergistically with Cbp1.

### Chitosan accumulation in the tips of germ tubes is important in inducing appressorium formation

**Figure 4:**
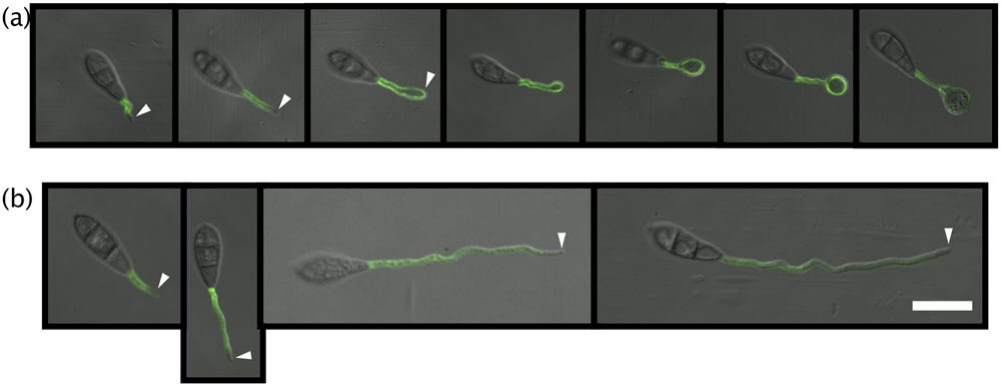
Time lapse of OGA^488^ fluorescence from conidia during germ tube elongation. OGA^488^ fluorescence was observed in the WT (**a**) and Δ*cbp1* mutant (**b**). Conidia were placed on PHOB-PC for 1–6 hpi and stained with OGA^488^. We estimated germ tube length as an indicator of growth stage. Arrowheads show the tips of germ tubes. Bar = 20 μm.

Cbp1 seemed to be involved in inducing appressorium formation, so we focused on chitosan in germ tubes. We stained chitosan at the cell surface using the chitosan-specific dye OGA^488^ ^33^ during germ tube elongation. OGA^488^ is an oligogalacturonate coupled to Alexa Fluor 488. OGA^488^ retains its natural affinity. It is impossible to observe the same conidium in consecutive sections because of the staining and washing procedures. Thus, in this study, we measured germ tube length as an indicator of the conidial development stage. In the wild type strain, fluorescence from OGA^488^ accumulated in the tips of germ tubes during germ tube elongation (Fig. 4(a) arrowhead). In contrast, very little fluorescence was detected in the tips of germ tubes of the Δ*cbp1* mutant (Fig. 4(b), arrowhead). This result showed that chitosan was accumulated in the tips of germ tubes when *M. oryzae* was in the induction phase of appressorium formation.

## Discussion

It was previously reported that Cbp1 is involved in signalling pathways associated with appressorium formation^16^, but how it behaves and the important role it plays were not clear. In this study, we investigated how Cbp1 works in forming an appressorium, focusing on the putative CDA activity of Cbp1.

We found that Cbp1 possesses CDA activity and that substitution mutations in the CDA active site cause a decrease in this activity. This is the first report of experimentally observed CDA activity in *M. oryzae*. In the Δ*cbp1* mutant, *CBL4* was upregulated but did not compensate for the function of Cbp1. However, upregulation of all *CBL*s was induced by the addition of HDD, which also induced appressorium differentiation at an early phase, as in the wild type. These results indicated that Cbp1 plays a main role in appressorium formation by converting chitin into chitosan during the early phase of appressorium differentiation. Furthermore, *CBL6* was upregulated in both the wild type and Δ*cbp1* by HDD addition, but the rate of increase was greater in the wild type than in the Δ*cbp1* mutant. We reasoned that expression of *CBL6* during the early phase of appressorium differentiation might therefore be affected by Cbp1.

The relationship between CDA activity and pathogenicity has been investigated in some other pathogens. The pathogenic fungus *Cladosporium fulvum*, which causes leaf-mould disease in tomatoes, has an effector known as Ecp6^34^. Ecp6 contains LysM domains that have been shown to bind chitin. By binding chitin, LysM suppresses the elicitor activity of chitin and pathogen-associated molecular pattern triggered defence systems^35^.

Geoghegan and Gurr^18^ proposed that Cbp1 does not function only in avoiding the host defence system because it seems to function as a CDA and is also involved in signalling. They showed that the presence of chitosan or the addition of exogenous chitosan at the cell surface of *M. oryzae* induced appressorium formation. However, they did not show the direct enzymatic detection of CDA activity of Cbp1.

In the case of phytopathogenic fungi, chitin may have become well masked during plant–microbe interactive co-evolution to avoid it acting as an elicitor and may no longer be a major defence target of plants. Our data suggested *M. oryzae* accumulates chitosan at the tips of germ tubes during induction of appressoria. We inferred that the amount of chitosan in the tips of germ tubes was sufficient and that the production of a sufficient amount of chitosan mainly depends on Cbp1 activity in the early phase of appressorium formation.

If the CDA activity of Cbp1 plays an important role in appressorium differentiation, other CDAs should be able to compensate for the loss of Cbp1. However, so far, we have not found any evidence that other CDAs can compensate for the Δ*cbp1* mutation. We are now focusing on the localization of Cbp1 in detail. Cbp1 has a Ser/Thr rich cluster and a putative GPI-anchoring signal as noted previously. These features strongly suggest that Cbp1 localizes to the surface of the cellular membrane or cell wall. Aside from Cbp1 and Cbl6, the other CDAs seem to be simple secreted proteins. We hypothesize that *M. oryzae* perceives signals from CDA activity with spatial-temporal exactness in the tips of germ tubes to activate the appressorium formation signalling pathway. Through these signals, *M. oryzae* may detect the conversion of chitin to chitosan under appropriate circumstances and conduct appressorium differentiation.

## Methods

### Fungal strains and culture conditions

*M. oryzae* wild type strain P2 was maintained as a stock culture in our laboratory and used as a wild type control throughout this study. Fungal strains were grown on oatmeal agar medium containing 5.0% oatmeal (Quaker Oats Company, Chicago, IL, USA), 0.5% sucrose (Nacalai Tesque, Kyoto, Japan) and 1.5% agar (Wako Pure Chemical Industries, Ltd., Osaka, Japan) at 28 °C. Conidiation was induced under BLB lamps (FL20S, Toshiba Co. Ltd, Tokyo, Japan) after removing the aerial hyphae with a sterilized brush. After 2–3 days of incubation under BLB lamps, the conidia were brushed off into distilled water. Mycelia for genomic DNA and RNA isolation and protoplast preparation were grown in YG liquid medium (0.5% yeast extract (Nacalai Tesque) and 2.0% glucose (Nacalai Tesque)) with shaking at 28 °C.

### CBP1 point mutation

A KOD^+^ site directed mutagenesis kit (TOYOBO Co., Ltd., Osaka, Japan) was used to introduce point mutations. The 5′ termini of the primers were methylated using T4 Polynucleotide Kinase (Takara Bio Inc., Shiga, Japan) and pKS01 was amplified with the methylated primers using PCR (95 °C, 2 min → [95 °C, 20 sec; 65 °C, 30 sec; 68 °C, 7 min] × 5 cycles). The CBP1_D161Af and CBP1_D161Ar primer pair was used to convert the first aspartate residue in the (a) domain (Fig. 1), CBP1_D162Af and CBP1_D161Ar for the second aspartate residue in the (a) domain (Fig. 1), and CBP1_R258Af and CBP1_R258Ar for the arginine in the (b) domain (Fig. 1). We transformed *Escherichia coli* DH5α with the PCR product, which was digested by DpnI and ligated. Point mutations in *M. oryzae* were introduced using this vector.

### Heterologous expression in *E. coli* and CDA activity assay

Cbp1 and inactive Cbp1 were amplified by EcoRI_CBP1_F and SalI_CBP1_R and introduced into the pCold I vector (TakaRa) for transformation of *E. coli* Rosetta (F-, ompT, hsdSB (rB−mB−), gal, dcm (DE3) pRARE (CamR)) (Novagen, Madison, WI, USA). The primers used in this study are listed in Table S1. After incubation in LBA liquid medium (0.1% NaCl (Nacalai Tesque), 0.1% tryptone (Nacalai Tesque), 0.05% yeast extract, and 100 µg/mL ampicillin sodium salt (Nacalai Tesque)) at 37 °C, 1.0 mM IPTG was added and the culture was incubated for 24 h at 15 °C. The *E. coli* cells were then harvested by centrifugal separation and ultrasonication. Cbp1 was collected as an insoluble precipitate, because of inclusion body formation. The inclusion bodies were washed three times by ultrasonic fragmentation in washing buffer (20 mM Tris-HCl (pH 8.0; Nacalai Tesque), 2 mM MgCl_2_ (Nacalai Tesque), 2 units/mL benzonase endonuclease (Novagen) and 10 µg/mL lysozyme (Wako)). After washing, the precipitate was suspended with sterilized water to give a crude extract derived from 1 × 10^7^ total bacterial count per µL. CDA activity was measured by the MBTH method following the protocol previously reported^36^ and glycol chitin was synthesized from glycol chitosan (Wako) as previously reported^37^. Briefly, 100 µL of 50 mM sodium tetraborate buffer (pH 8.5) (Nacalai Tesque), 100 µL of 0.1% glycol chitin and 50 µL of crude extract sample were mixed and incubated for 30 min at 37 °C. Then, 250 µL of 2 N H_2_SO_4_ was added to stop the enzyme reaction. After adding 250 µL of 5% NaNO_3_ and incubating for 5 min at RT, 250 µL of 12.5% ammonium sulfate was added and the mixture was incubated for 15 min at RT. Then, 250 µL of 0.5% MBTH (Tokyo Chemical Industry Co., Ltd., Tokyo, Japan) was added and the samples were boiled for 3 min and rapidly cooled. After that, 25 µL of 5% FeCl_3_ was added to develop a colour and the samples were incubated for 30 min. CDA activity was measured by the absorbance at 650 nm.

### Extraction of genomic DNA and transformation of *M. oryzae*

*M. oryzae* hyphae were incubated in 30 mL of YG liquid medium at 28 °C, 150 rpm for 2 days and then inoculated into 100 mL YG liquid medium and kept at 28 °C, 150 rpm for 1 day. Then, the fungal mycelium samples were harvested by centrifugation at 2000 × *g* for 10 min and freeze-fractured. Extraction of genomic DNA from the fungal mycelia was performed following a previously described method^38^. Protoplasting and transformation of *M. oryzae* with the selectable markers *BSD* or *HPH* were performed as described previously^39^.

### Appressorium formation assay

Conidia were harvested from 5 to 7-day-old cultures on oatmeal agar medium and adjusted to 3 × 10^4^ conidia per mL with sterile distilled water. Drops of the conidia suspension (20 µL) were placed on the surface of hydrophobic polyvinyl chloride (PHOB-PC) (Thermo Fisher Scientific, Inc., Waltham, MA, USA). The appressorium formation assays were performed at least three times with triplicate slides. The percentage of germinated conidia forming appressoria was observed by microscope at 6 h post inoculation (hpi). The appressorium formation rates were calculated as the number of germinated conidia with appressoria divided by the number of germinated conidia and the data were analysed statistically by Student’s *t*-test. Chemicals were used as appressorium formation inducers; 10 µM 1,16-hexadecanediol (HDD, (Tokyo Chemical Industry Co., Ltd.)) suspended in 0.1% ethanol.

### Fluorescence analysis

To observe chitosan, appressorium formation was induced and the conidia with appressoria were stained with OGA^488^. Staining with OGA^488^ was performed as described previously^33^. After incubation, the samples were washed twice with 100 µL of 25 mM MES buffer, pH 5.7. Then, droplets of OGA^488^ were diluted 2000-fold with 25 mM MES buffer, pH 5.7 and kept in the dark for 20 min. The samples were washed twice with 100 µL MES buffer before observation. We used an LSM 5 EXCITER (Carl Zeiss AG, Oberkochen, Germany), Ar laser (488 nm), HFT 488 beam splitter and 505-530 suppression filter for fluorescence observations.

### Homology search

Protein sequences were obtained from National Center for Biotechnology Information (http://www.ncbi.nlm.nih.gov). We used BLAST^40^ for homology searches and CLUSTALW^41^ to compare amino acid sequences.

### RNA extraction and real-time PCR

To obtain RNA from the germling stage, we induced appressorium formation on hydrophobic polycarbonate plates (SANPLATEC Co., Ltd, Osaka, Japan) instead of PHOB-PC. At 3 hpi, when some conidia were germinating, and at 6 hpi, when some conidia formed appressoria in the wild type, the germlings on polycarbonate plates were frozen with liquid nitrogen and continuously lyophilized in a vacuum freeze dryer FDU-2110 (TOKYO RIKAKIKAI Co., Ltd, Tokyo, Japan). Then, we collected the germinated conidia from the hydrophobic polycarbonate plates using a razor. RNA samples were extracted from the conidia using RNAzol (Cosmo Bio Co., Ltd., Tokyo, Japan) according to the manufacturer’s protocol. To synthesize first-strand cDNA from the RNA, we used ReverTra Ace (TOYOBO Co., Ltd.) following the protocols of the kit. Real-time PCR was performed using SYBR green on a 7300 Real-Time PCR instrument (Thermo Fisher Scientific, Inc.). The expression levels of *CBP1* and the *CBL*s were normalized by the expression levels of internal standard, *HPRT* (hypoxanthine phosphoribosyl transferase: *MGG_10052*). We used the primers CBP1rt14_S and CBP1rt14_AS for *CBP1* detection, CBL1_up and CBL1_down for *CBL1*, CBL2_up and CBL2_down for *CBL2*, CBL3_up3 and CBL3_down3 for *CBL3*, CBL4_up and CBL4_down for *CBL4*, CBL5_up and CBL5_down for *CBL5*, CBL6_up2 and CBL6_down2 for *CBL6*, and HPRT up and HPRT down for *HPRT*.

## Electronic supplementary material

Supplymentary information
