# Supplementary material for: Chitin-deacetylase activity induces appressorium differentiation in the rice blast fungus Magnaporthe oryzae
Source: Sci Rep. 2017 Aug 29;7:9697. doi: 10.1038/s41598-017-10322-0 (PMC5575296; doi:10.1038/s41598-017-10322-0)
Supplement: Supplementary file 1 — Supplymentary information [file 41598_2017_10322_MOESM1_ESM.pdf]

Title: Chitin-deacetylase activity induces appressorium differentiation in the rice blast fungus *Magnaporthe oryzae*

Authors:

Misa Kuroki[1], Kana Okauchi[1], Sho Yoshida[1], Yuko Ohno[1], Sayaka Murata[1], Yuichi Nakajima[2], Akihito Nozaka[1], Nobukiyo Tanaka[1], Masahiro Nakajima[1], Hayao Taguchi[1], Ken-ichiro Saitoh[3], Tohru Teraoka[4], Megumi Narukawa[1], Takashi Kamakura[1]*

Supplementary Table S1 Primers used in this study.

name	sequence (5' -> 3')
CBP1rt14_S	CGGACAACTTCGTCCACAGTT
CBP1rt14_AS	CCGAGGTGCTCGTTTTGCT
CBL1_up	CTGTGGTCGAAAAGCGTGCGGGAATC
CBL1_down	GTACTGGAACAAGCAG
CBL2_up	CATCTAGGCGAGCAGACACATC
CBL2_down	CGGCTCAGCAGGTCCAGC
CBL3_up3	CCACGATAACGAGGAAGCCACT
CBL3_down3	CCAGTCTGCGGATTGCGGTAAAA
CBL4_up	CGATGTTAGGGCCGGATTTG
CBL4_down	CTTCGCTCTACATTGCTG
CBL5_up	GATTGCCGTCTCCTTTGTC
CBL5_down	CTGTAGTCGCGCTCGTTGATGC

CBL6_up2	CTTGCCGTTGAGGAAGAAGGT
CBL6_down2	CAGCTGAAGTCTTGCGTGGA
HPRT up	CGTCGAGGCCGCCAGGGTCAA
HPRT down	CCACGGGTAGCAGATCCAAACATCC
CBP1_D161Af	CTGACCTTTGCCGACGGTCCATACAA
CBP1_D161Ar	GGCAATATCACCCTGGGTAACACA
CBP1_D162Af	CTGACCTTTGACGCCGGTCCATACAA
CBP1_R258Af	CCCGACTTACATGGCGCCACCTTAC
CBP1_R258Ar	AAGTATCCAATGATGTCAGCAAGAG
EcoRI_CBP1_F	AAAGAATTCATGAAGTGGTTGTCACTTGCCC
SalI_CBP1_R	AAAGTCGACCTACCATAAACAGAACCCG

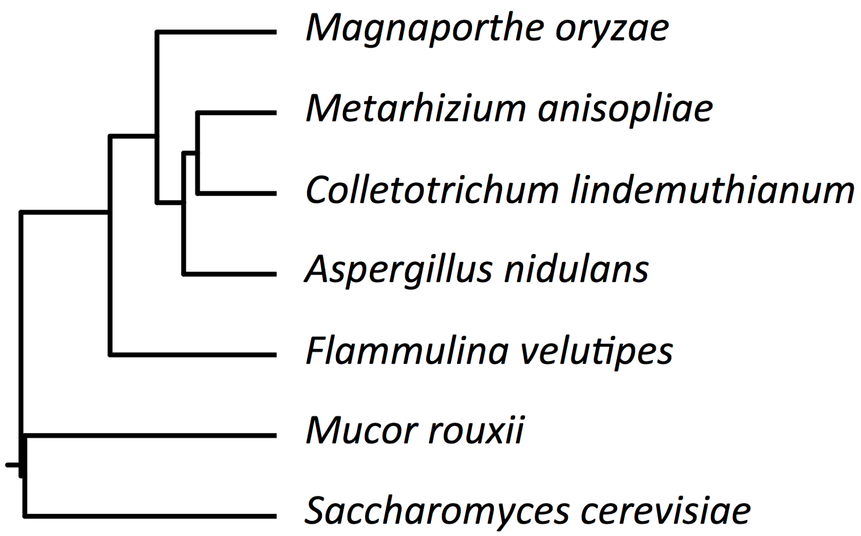

Supplementary Fig. S1 Phylogenetic relationships of *M. oryzae* Cbp1 to CDAs in other species.

The amino acid sequences of the CDA domains in representative species were analysed by the Wagner parsimony method weighted according to Felsenstein's amino acid substitution rules.

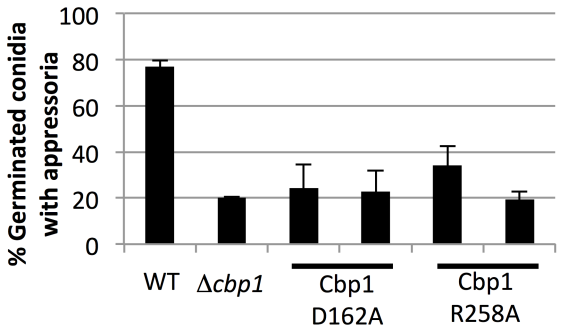

Supplementary Fig. S2 Effect of amino acid substitutions in Cbp1 on appressorium formation at 6 hpi.

Appressorium formation rates were scored at 6 hpi on PHOB-PC. The experiments was performed in triplicate for each sample and repeated three times. Appressorium formation rates were calculated by dividing the number of conidia with appressoria by the number of germinated conidia. Cbp1 D162A has point mutation changing the second aspartate residue in the (a) region (Fig. 1) to alanine. Cbp1 R258A has point mutation changing the arginine in the (b) region (Fig. 1) to alanine. The appressorium formation rates of Cbp1 D162A and Cbp1 R258A were not different from that of Δ*cbp1* (Student's *t*-test). Error bars indicate standard error.

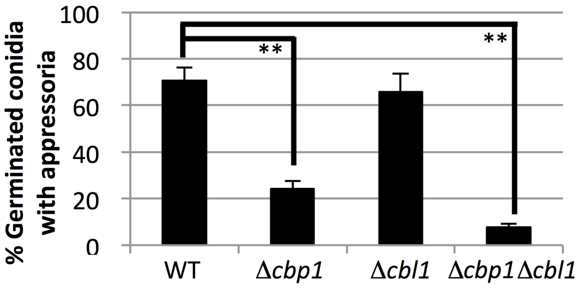

Supplementary Fig. S3 Appressorium formation rates of the Δ*cbl1* single mutant and Δ*cbp1*Δ*cbl1* double mutant strains at 6 hpi.

Appressorium formation rates were scored at 6 hpi on PHOB-PC. The experiment was performed in triplicate for each sample and repeated three times. Appressorium formation rates were calculated by dividing the number of conidia with appressoria by the number of germinated conidia. ** $p < 0.01$ (Student's t-test) compared with WT. Error bars indicate standard error.
